# Develop prediction model to help forecast advanced prostate cancer patients’ prognosis after surgery using neural network

**DOI:** 10.3389/fendo.2024.1293953

**Published:** 2024-03-21

**Authors:** Shanshan Li, Siyu Cai, Jinghong Huang, Zongcheng Li, Zhengyu Shi, Kai Zhang, Juan Jiao, Wei Li, Yuanming Pan

**Affiliations:** ^1^ Department of Clinical Laboratory, The Seventh Medical Center of Chinese PLA General Hospital, Beijing, China; ^2^ Cancer Research Center, Beijing Chest Hospital, Capital Medical University/Beijing Tuberculosis and Thoracic Tumor Research Institute, Beijing, China; ^3^ Dermatology Department, General Hospital of Western Theater Command, Chengdu, Sichuan, China; ^4^ Department of Biochemistry, School of Medicine/Key Laboratory of Xinjiang Ministry of Education, Shihezi University, Shihezi, Xinjiang, China; ^5^ Urinary Surgery Department, The First People’s Hospital of Ziyang, Ziyang, Sichuan, China; ^6^ Chengdu Eighth People’s Hospital, Chengdu, Sichuan, China; ^7^ General Department, Beijing Chest Hospital, Capital Medical University/Beijing Tuberculosis and Thoracic Tumor Research Institute, Tongzhou District, Beijing, China

**Keywords:** prediction model, prostate cancer, prognosis, surgery, neural network, deep learning

## Abstract

**Background:**

The effect of surgery on advanced prostate cancer (PC) is unclear and predictive model for postoperative survival is lacking yet.

**Methods:**

We investigate the National Cancer Institute’s Surveillance, Epidemiology, and End Results (SEER) database, to collect clinical features of advanced PC patients. According to clinical experience, age, race, grade, pathology, T, N, M, stage, size, regional nodes positive, regional nodes examined, surgery, radiotherapy, chemotherapy, history of malignancy, clinical Gleason score (composed of needle core biopsy or transurethral resection of the prostate specimens), pathological Gleason score (composed of prostatectomy specimens) and prostate-specific antigen (PSA) are the potential predictive variables. All samples are divided into train cohort (70% of total, for model training) and test cohort (30% of total, for model validation) by random sampling. We then develop neural network to predict advanced PC patients’ overall. Area under receiver operating characteristic curve (AUC) is used to evaluate model’s performance.

**Results:**

6380 patients, diagnosed with advanced (stage III-IV) prostate cancer and receiving surgery, have been included. The model using all collected clinical features as predictors and based on neural network algorithm performs best, which scores 0.7058 AUC (95% CIs, 0.7021-0.7068) in train cohort and 0.6925 AUC (95% CIs, 0.6906-0.6956) in test cohort. We then package it into a Windows 64-bit software.

**Conclusion:**

Patients with advanced prostate cancer may benefit from surgery. In order to forecast their overall survival, we first build a clinical features-based prognostic model. This model is accuracy and may offer some reference on clinical decision making.

## Background

Prostate cancer (PC) is the second-most common solid organ malignancy globally and the most prevalent solid organ malignancy in males in the United States (1). In Western nations, the second-most prominent cause of men’s cancer-related mortality is also PC, and more than 30,000 men die from it in the United States (2). Race, age, family history, obesity, and other conditions are mainly risk factors for PC (3, 4). Usually, PC patients with T3-T4, prostate-specific antigen (PSA) ≥ 20 ng/ml, lymph node or distant site metastasis have the potential for being diagnosed with advanced PC.

Advanced PC is typically regarded as incurable. On one hand, since Charles Huggins initially observed the impact of androgen deprivation therapy (ADT) on metastatic PC patients, inhibition of androgen receptor signaling with ADT has been the basis of therapy for metastatic PC. ADT has involved several types, like surgical castration or pharmacological castration. However, despite the fact that ADT provides about 1-2 years’ remissions in the majority of patients, PC can grow resistant, called metastatic castration-resistant PC (5). On the other hand, traditionally, advanced PC is still dominated by ADT treatment, and radical prostatectomy (RP) is rarely the first option. The primary cause may be that the presence of tumor extension into the rhabdosphincter, rectal wall, and seminal vesicles usually implies a poor prognosis and is often accompanied by fatal surgical complications (6, 7). However, with the improvement and refinement of surgical technology, particularly the introduction of robot-assisted radical prostatectomy (RALP), the prognosis of advanced PC is steadily improving, and the rate of surgical complications may also be handled (7, 8). In recent years, cytoreductive prostatectomy (CP) has gradually attracted attention. Some evidence suggests a feasible role for CP in metastatic PC (9–11). Axel Heidenreich et al. observed that advanced PC patients responding well to neoadjuvant androgen deprivation therapy had a better progression-free survival (PFS) (38.6 vs 26.5 months, P = 0.032) after CP than control group (9). These findings imply that surgery might be a novel and effective treatment option for advanced PC. However, there is no consensus on which patients are appropriate or how to predict their outcome.

In this study, we investigate the National Cancer Institute’s Surveillance, Epidemiology, and End Results (SEER) database for records regarding PC patients with staged III-IV and undergoing surgery, and create a neural network prediction model to estimate their postoperative survival. We then package the model into a software, which is convenient for clinicians to use and decision-making assistance.

## Methods

### Patients and datasets

**Figure 1:**
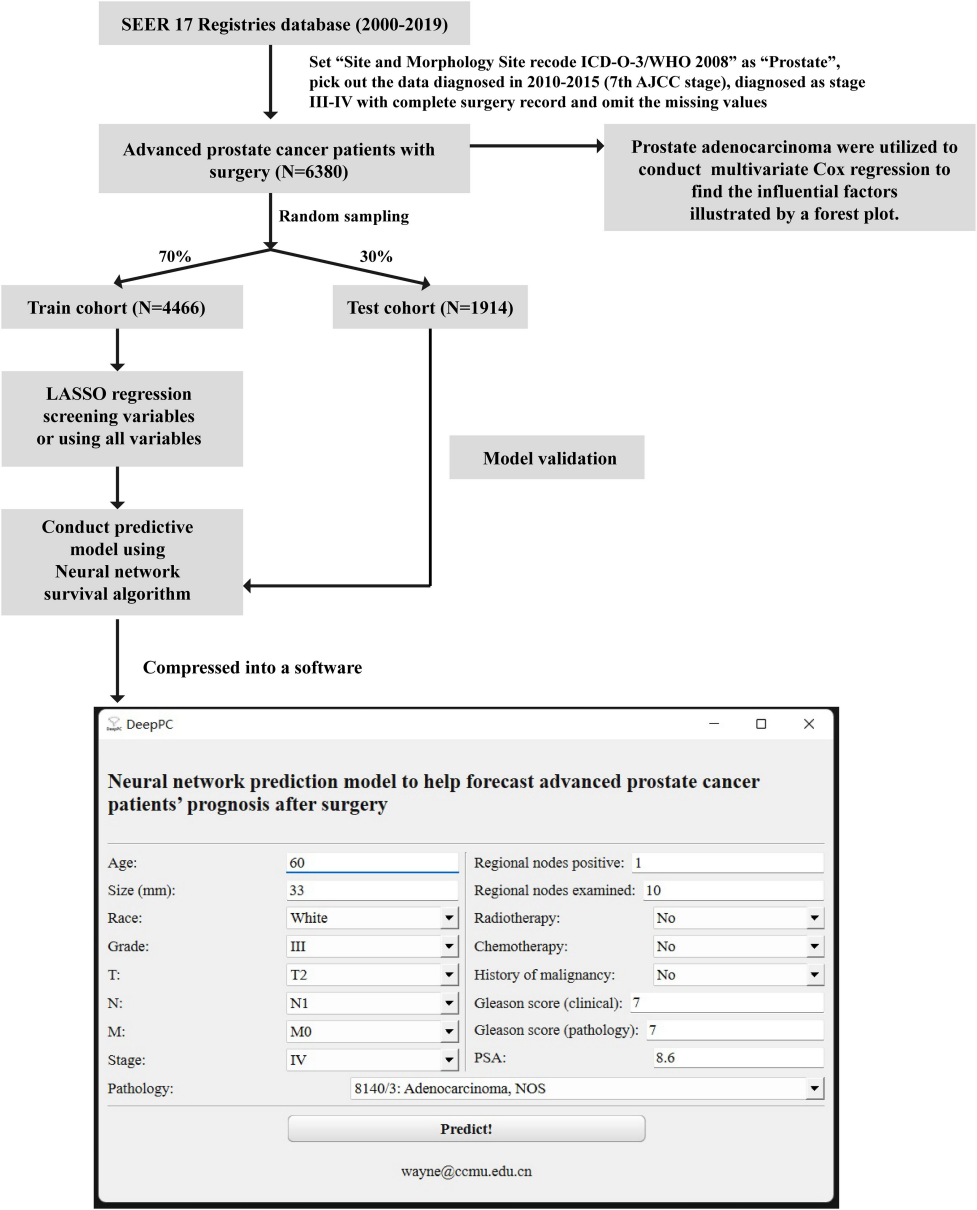
Flow chart of this study. SEER, Surveillance, Epidemiology, and End Results database; AJCC, American Joint Committee on Cancer; LASSO, least absolute shrinkage and selection operator; PSA, prostate-specific antigen.

Retrieving with SEER*Stat (8.4.0), we utilize the 17 Registries database (2000–2019), which covers approximately 26.5% of the U.S. population, and set “Site and Morphology. Site recode ICD-O-3/WHO 2008” as “Prostate” to get the raw data of prostate cancer. Raw data are filtered to reserve patients diagnosed in 2010-2015 years for they containing the detailed 7^th^ American Joint Committee on Cancer (AJCC) stage and confirmed as stage III-IV with complete surgery records. Samples with missing values are omitted, and 5 samples are taken out due to their contradictory records about lymphatic metastasis. 6380 samples are adopted finally. Then all patients are divided into train cohort (70% of total) and test cohort (30% of total) by random sampling. Train cohort is used to conduct survival models, validated by its own and test cohort. According to SEER’s criteria, tumor diameters exceeding 989mm are still recorded as 989mm, and patients over the age of 100 are still documented as 100 (**Figure 1**).

The SEER program registries routinely collect demographic and clinic data, and the mortality data reported by SEER were provided by the National Center for Health Statistics, according to SEER website. The 17 Registries database (2000-2019) is submitted in November 2021, and the follow-up cut-off date is December 31, 2019, according to SEER description manual. We have signed the SEER Research Data Use Agreement to acquire access.

### Data cleaning and feature engineering

Usually more predictive variables show a better performance, so we collect them as much as possible. According to clinical experience, age, race, grade, pathology, T, N, M, stage, size, regional nodes positive, regional nodes examined, surgery, radiotherapy, chemotherapy, history of malignancy, clinical Gleason score (composed of needle core biopsy or transurethral resection of the prostate specimens), pathological Gleason score (composed of prostatectomy specimens) and PSA are the potential predictive variables. At first, all clinical features above are used to conduct models, with evaluated fitting and overfitting in both train and test cohorts.

Then, considering the potential multicollinearity among these variables (though sometimes not considered in neural network model), we apply least absolute shrinkage and selection operator (LASSO) regression to screen clinical features mentioned above. The key to LASSO regression is to allocate an appropriate lambda value, which is confirmed by a 5-fold cross validation and the minimum one is adopted. The clinical features with a non-zero coefficient in LASSO regression (short for LASSO variables) are taken out to build prognostic model. Only the train cohort is used in this process, and R package glmnet is used to achieve work above.

### Survival model training and evaluation

All data are separated into two parts, train cohort for LASSO regression and conducting models, and test cohort for further validation. Some variables (T and M) are merged, although they are shown specifically on the baseline table.

Using the pytorch platform based on python 3.9.7, we construct a deep learning survival model to predict overall survival (OS) probability of the PC patients. The deep learning survival model contained input layers (the clinical features), activation layers (convert the computing results to nonlinear ones), drop out layers (silence some neurons randomly to avoid overfitting) and batch normalization layers (ensure that the mean and variance of the input variables are fixed within a certain range to improve model performance). We turn on the early stopping function, which can end training automatically when model’s performance gets no improvement after several rounds of trainings (set as 30 rounds here). Batch size training is enabled and 512 samples are used each time. Adam is designated as optimizer with 0.05 learning rate. Numerical clinical features are normalized (subtract the mean and divide by the standard deviation) and categorical clinical features are transformed into number encodings before training. Python package pandas, numpy, pycox, matplotlib, lifelines and scikit-learn assist us with the above process.

The traditional CPH model has been built too, to make a contrast, with the help of python package lifelines. All models are conducted using both all clinical features collected this time or LASSO filtered ones.

The main evaluation indicator is area under receiver operating characteristic curve (AUC). An AUC closer to 1.0 reflects the model perfect in predicting, while a model scoring 0.5 AUC tends to random guess. We evaluate models in both train and test cohorts, reporting the mean AUC and 95% confidence interval (CIs) by Bootstrap.

The neural network is compressed as a graphical user interface (GUI) software for clinicians to use finally.

### Survival analysis

All data both train and test cohorts are finally employed to conduct Cox proportional hazard (CPH) regression, revealing the hazard ratio (HR) and 95% CIs to discover influential factors of advanced prostate adenocarcinoma (8140/3) after surgery. A forest plot is drawn to visualize results above with the use of R package ezcox, survival and survminer.

### Statistical analysis

This study is analyzed with R software. The comparison between train cohort and test cohort is assessed using Student’s t or Mann-Whitney U test for continuous variables, Chi-square test for categorical variables. P < 0.05 of two-sided is considered statistically significant.

This research is conducted in accordance with the Declaration of Helsinki. This retrospective cohort study uses data from the publicly available SEER database, patients’ information has been anonymized and not traceable. And the data submitters have gotten informed consent from participants and obtained the ethical permission. Given that, this research is exempted from ethical applications and written consent.

## Results

### Clinical characteristics

A total of 6380 patients, diagnosed with advanced (stage III-IV) PC and receiving surgery, have been included. After random sampling, 4466 (70% of total) in train cohort and 1914 (30% of total) in test cohort. The detailed clinical information is displayed in **Table 1**. Two cohorts have no significant difference in clinical features. The mean age is 63.28 years old in train cohort and 63.1 years old in test cohort. Most patients are white and diagnosed with grade III in two cohorts. Adenocarcinoma is the most common pathology type. Most patients are staged T3a, N0, M0 or stage III. The median tumor diameter is 23 mm in train cohort and 22 mm in test cohort. The median regional nodes positive is 0 and median regional nodes examined is 7 in both two cohorts. Most patients got no radiotherapy or chemotherapy, and had no history of malignancy. The median Gleason score is 7 (either clinical or pathology) in two cohorts. The median PSA is 8 ng/ml in train cohort and 7.8 ng/ml in test cohort. The median survival time is 75 months in train cohort and 73 months in test cohort. Most patients survive in both two cohorts.

**Table 1:** Clinical information of two cohorts.

	Train cohort	Test cohort	Statistical method	P value
	(N=4466)	(N=1914)		
	No. (%)			
Age			Student’s t	0.3349
Mean (SD)	63.28 (6.84)	63.1 (7.08)		
Race			Chi-square	0.8640
White	3611 (80.86)	1542 (80.56)		
Black	410 (9.18)	173 (9.04)		
Other	445 (9.96)	199 (10.40)		
Grade			Chi-square	0.7252
I	22 (0.49)	13 (0.68)		
II	1079 (24.16)	472 (24.66)		
III	3354 (75.10)	1423 (74.35)		
IV	11 (0.25)	6 (0.31)		
Pathology			Chi-square	0.3535
8140/3: Adenocarcinoma, NOS	4399 (98.50)	1880 (98.22)		
8201/3: Cribriform carcinoma, NOS	2 (0.04)	0 (0)		
8246/3: Neuroendocrine carcinoma, NOS	2 (0.04)	0 (0)		
8255/3: Adenocarcinoma with mixed subtypes	11 (0.25)	6 (0.31)		
8480/3: Mucinous adenocarcinoma	5 (0.11)	4 (0.21)		
8481/3: Mucin-producing adenocarcinoma	1 (0.02)	1 (0.05)		
8490/3: Signet ring cell carcinoma	0 (0)	2 (0.10)		
8500/3: Infiltrating duct carcinoma, NOS	29 (0.65)	16 (0.84)		
8550/3: Acinar cell carcinoma	14 (0.31)	5 (0.26)		
8574/3: Adenocarcinoma with neuroendocrine differentiation	3 (0.07)	0 (0)		
T			Chi-square	0.9349
T2	11 (0.25)	2 (0.10)		
T2a	7 (0.16)	3 (0.16)		
T2b	6 (0.13)	4 (0.21)		
T2c	90 (2.02)	37 (1.93)		
T3	10 (0.22)	6 (0.31)		
T3a	2774 (62.11)	1181 (61.70)		
T3b	1506 (33.72)	654 (34.17)		
T4	62 (1.39)	27 (1.41)		
N			Chi-square	0.1863
N0	3747 (83.90)	1631 (85.21)		
N1	719 (16.10)	283 (14.79)		
M			Chi-square	0.1256
M0	4431 (99.22)	1905 (99.53)		
M1a	5 (0.11)	2 (0.10)		
M1b	29 (0.65)	5 (0.26)		
M1c	1 (0.02)	2 (0.10)		
Stage			Chi-square	0.0897
III	3686 (82.53)	1613 (84.27)		
IV	780 (17.47)	301 (15.73)		
Size			Wilcoxon signed-rank	0.1028
Median (IQR)	23 (17, 32)	22 (17, 30)		
Regional nodes positive			Wilcoxon signed-rank	0.2139
Median (IQR)	0 (0, 0)	0 (0, 0)		
Regional nodes examined			Wilcoxon signed-rank	0.4381
Median (IQR)	7 (3, 12)	7 (4, 12)		
Radiotherapy			Chi-square	0.3138
No	3613 (80.9)	1569 (81.97)		
Yes	853 (19.1)	345 (18.03)		
Chemotherapy			Chi-square	0.1210
No	4423 (99.04)	1903 (99.43)		
Yes	43 (0.96)	11 (0.57)		
History of malignancy			Chi-square	0.7347
No	4164 (93.24)	1789 (93.47)		
Yes	302 (6.76)	125 (6.53)		
Gleason score (clinical)			Wilcoxon signed-rank	0.6437
Median (IQR)	7 (7, 8)	7 (7, 8)		
Gleason score (pathology)			Wilcoxon signed-rank	0.3687
Median (IQR)	7 (7, 8)	7 (7, 8)		
PSA			Wilcoxon signed-rank	0.5917
Median (IQR)	8 (5.6, 13.4)	7.8 (5.6, 13.3)		
Survival time			Wilcoxon signed-rank	0.0592
Median (IQR)	75 (58, 96)	73 (57, 95)		
Dead			Chi-square	0.5402
No	4015 (89.90)	1711 (89.39)		
Yes	451 (10.10)	203 (10.61)		

SD, standard deviation. Grade I, well differentiated; Grade II, moderately differentiated; Grade III, poorly differentiated; Grade IV, undifferentiated, anaplastic. NOS, not otherwise specified. IQR, inter-quartile range. Gleason score (clinical), composed of needle core biopsy or transurethral resection of the prostate specimens. Gleason score (pathology), composed of prostatectomy specimens. PSA, prostate-specific antigen.

### Predictive variables

We conduct models using all clinical features at first. Then LASSO regression is used to discover non-zero coefficient variables and screen clinical features (**Supplementary Figure 1A**). Concrete coefficient values are exhibited on **Supplementary Table 1**. Finally, these clinical features are picked out: age, M, stage, chemotherapy, history of malignancy, clinical Gleason score, pathological Gleason score, which all above are short for LASSO variables. All variables and LASSO variables are both used to conduct models too, by neural network and CPH. Prior to training, numerical clinical features are standardized according to their mean and standard deviation (**Supplementary Table 2**), and categorical clinical characteristics are converted into number encodings (**Supplementary Table 3**).

### Model performance

LASSO variables are input, then a neural network is finished training after 35 epochs, according to the deep learning custom and tuning. The model has 0.6811 AUC (95% CIs: 0.6799-0.6849) in train cohort and 0.6779 AUC (95% CIs: 0.6740-0.6790) in test cohort (**Table 2**). The training curve has been saved in (**Supplementary Figure 1B**).

**Table 2:** The performance of conducted models.

	Cox proportional hazard model				Neural network survival model			
	Train cohort		Test cohort		Train cohort		Test cohort	
	AUC	95% CI	AUC	95% CI	AUC	95% CI	AUC	95% CI
LASSO vars	0.6657	0.6633-0.6686	0.6719	0.6653-0.6707	0.6811	0.6799-0.6849	0.6779	0.6740-0.6790
All vars	0.6639	0.6604-0.6657	0.6696	0.6678-0.6731	0.7058	0.7021-0.7068	0.6925	0.6906-0.6956

AUC, area under receiver operating characteristic curve. CI, confidence interval. LASSO vars, least absolute shrinkage and selection operator screened out the predictive variables, including age, M, stage, size, chemotherapy, history of malignancy, Gleason score clinical (composed of needle core biopsy or transurethral resection of the prostate specimens) and Gleason score pathology (composed of prostatectomy specimens).

ALL vars, all variables collected this study, including LASSO variables, race, grade, pathology, T, N, regional nodes positive, regional nodes examined, radiotherapy and PSA (prostate-specific antigen).

When it comes to all variables, a neural network is finished training after 36 epochs. And this model scores 0.7058 AUC (95% CIs, 0.7021-0.7068) in train cohort and 0.6925 AUC (95% CIs, 0.6906-0.6956) in test cohort (**Table 2**). The training curve has been saved in (**Supplementary Figure 1C**).

We also execute CPH regression to compare, using all variables and LASSO variables. The LASSO variables’ AUC is 0.6657 (95% CIs: 0.6633-0.6686) and 0.6719 (95% CIs: 0.6653-0.6707) in train and test cohort respectively. All variables get AUC of 0.6639 (95% CIs: 0.6604-0.6657) and 0.6696 (95% CIs: 0.6678-0.6731) in train and test cohort respectively. Overall speaking, neural network has a better performance than CPH (**Table 2**).

### Model’s further evaluation and compression

**Figure 2:**
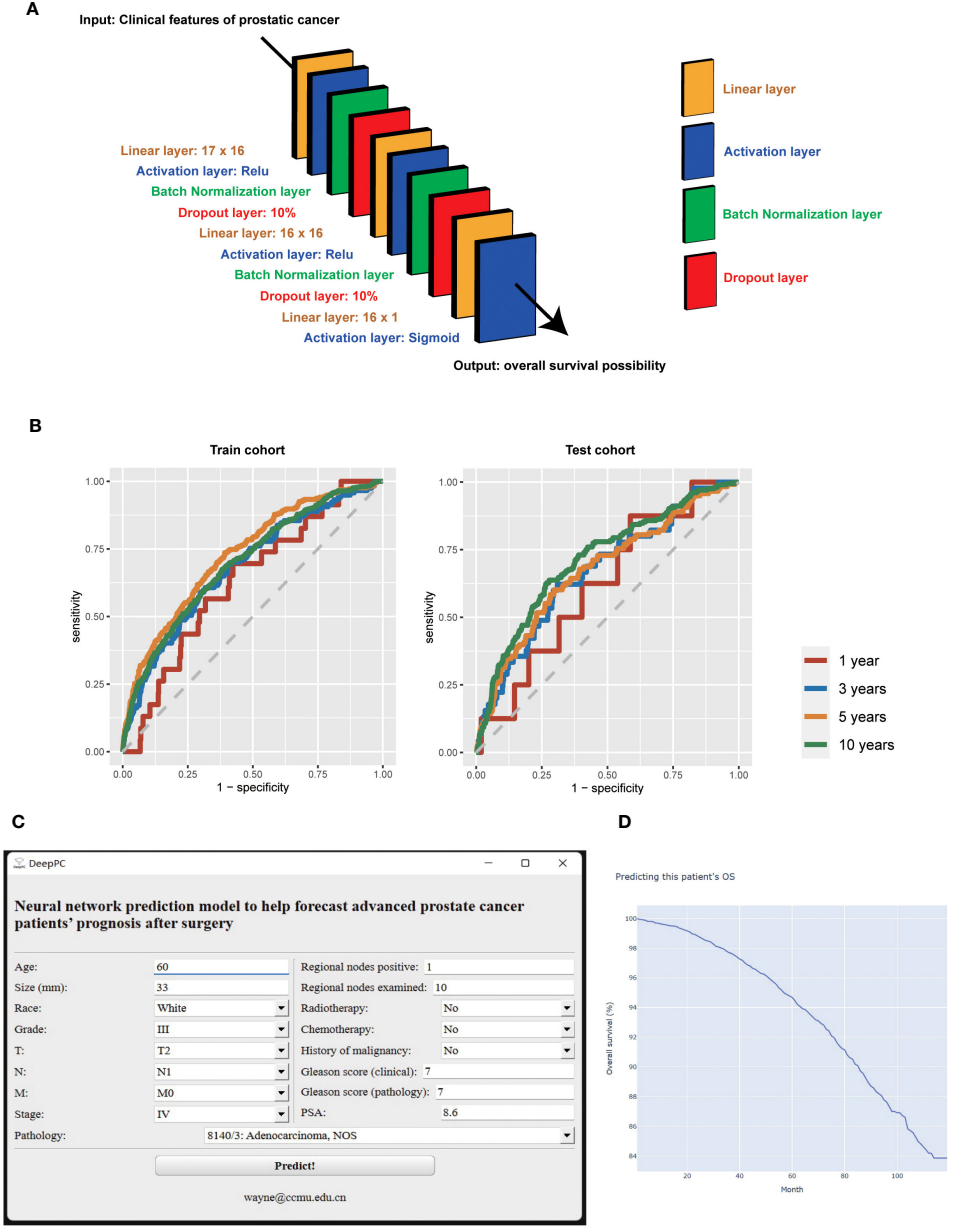
The structure **(A)**, receiver operating characteristic curves **(B)**, graphical user interface **(C)**, and computational results **(D)** of survival predictive tool for advanced prostate cancer patients after surgery (DeepPC). PSA, prostate-specific antigen.

Then we suggest the model calculating with all variables and based on neural network to serves as the survival predictive tool for advanced PC patients after surgery (DeepPC). The architecture of DeepPC is as follows: it has 10 layers, including a linear layer (17 x 16 nodes), an activation layer (Relu function), a batch normalization layer, a dropout layer (10%), a linear layer (16 x 16 nodes), another activation layer (Relu function), another batch normalization layer, another dropout layer (10%), a linear layer (16 x 1 nodes) and the final activation layer (Sigmoid transformation) (**Figure 2A**). The detailed parameters of DeepPC are stored in **Supplementary Figure 2**.

We then validate DeepPC further in its 1-, 3-, 5- and 10-years prediction of PC patients’ OS. For 1 year, DeepPC gets 0.6303 AUC (95% CIs, 0.5250-0.7357), 0.5764 specificity, 0.6957 sensitivity, 0.9973 negative predictive value (NPV) and 0.0084 positive predictive value (PPV) in train cohort, and 0.6210 AUC (95% CIs, 0.4381-0.8039), 0.4129 specificity, 0.8750 sensitivity, 0.9987 NPV and 0.0062 PPV in test cohort. For 3 years, DeepPC has 0.6834 AUC (95% CIs: 0.6331-0.7336), 0.7025 specificity, 0.5897 sensitivity, 0.9845 NPV and 0.0506 PPV in train cohort, and 0.6708 AUC (95% CIs: 0.5896-0.7519), 0.6907 specificity, 0.6222 sensitivity, 0.9870 NPV and 0.0462 PPV in test cohort. For 5 years, DeepPC shows 0.7294 AUC (95% CIs: 0.6974-0.7615), 0.6078 specificity, 0.7362 sensitivity, 0.9745 NPV and 0.1017 PPV in train cohort, and 0.6751 AUC (95% CIs: 0.6225-0.7276), 0.7021 specificity, 0.6017 sensitivity, 0.9641 NPV and 0.1172 PPV in test cohort. For 10 years, DeepPC scores 0.6990 AUC (95% CIs: 0.6733-0.7248), 0.6178 specificity, 0.6748 sensitivity, 0.9440 NPV and 0.1659 PPV in train cohort, and 0.7136 AUC (95% CIs: 0.6754-0.7517), 0.7216 specificity, 0.6373 sensitivity, 0.9434 NPV and 0.2145 PPV in test cohort. (**Table 3**) The receiver operating characteristic curves (ROC) of DeepPC in 1-, 3-, 5- and 10-years’ performance are illustrated in **Figure 2B**.

**Table 3:** The performance of neural network model in 1-, 3-, 5- and 10-years’ survival prediction.

	Train cohort				Test cohort			
	1 year	3 years	5 years	10 years	1 year	3 years	5 years	10 years
AUC	0.6303	0.6834	0.7294	0.6990	0.6210	0.6708	0.6751	0.7136
AUC 95% CI	0.5250-0.7357	0.6331-0.7336	0.6974-0.7615	0.6733-0.7248	0.4381-0.8039	0.5896-0.7519	0.6225-0.7276	0.6754-0.7517
Specificity	0.5764	0.7025	0.6078	0.6178	0.4129	0.6907	0.7021	0.7216
Sensitivity	0.6957	0.5897	0.7362	0.6748	0.8750	0.6222	0.6017	0.6373
NPV	0.9973	0.9845	0.9745	0.9440	0.9987	0.9870	0.9641	0.9434
PPV	0.0084	0.0506	0.1017	0.1659	0.0062	0.0462	0.1172	0.2145

AUC, area under receiver operating characteristic curve; CI, confidence interval; NPV, negative predictive value; PPV, positive predictive value.

We then compressed DeepPC into a GUI Windows software (**Figure 2C**). When age, race, grade, pathology, T, N, M, stage, size, regional nodes positive, regional nodes examined, surgery, radiotherapy, chemotherapy, history of malignancy, clinical Gleason score (composed of needle core biopsy or transurethral resection of the prostate specimens), pathological Gleason score (composed of prostatectomy specimens) and PSA of one prostate cancer patient are inputted, user can click “Predict!” button to launch the pre-trained DeepPC. After calculating, it will automatically open the user’s default browser to draw the patient’s survival curve (Kaplan-Meier curve) (**Figure 2D**). The curve is interactive. When the user hovers over, the specific month and survival probability will pop up automatically. We also keep the original python edition for easier processing when we need to predict the survival of PC patients in batches (**Supplementary Figure 3**).

### Survival analysis

**Figure 3:**
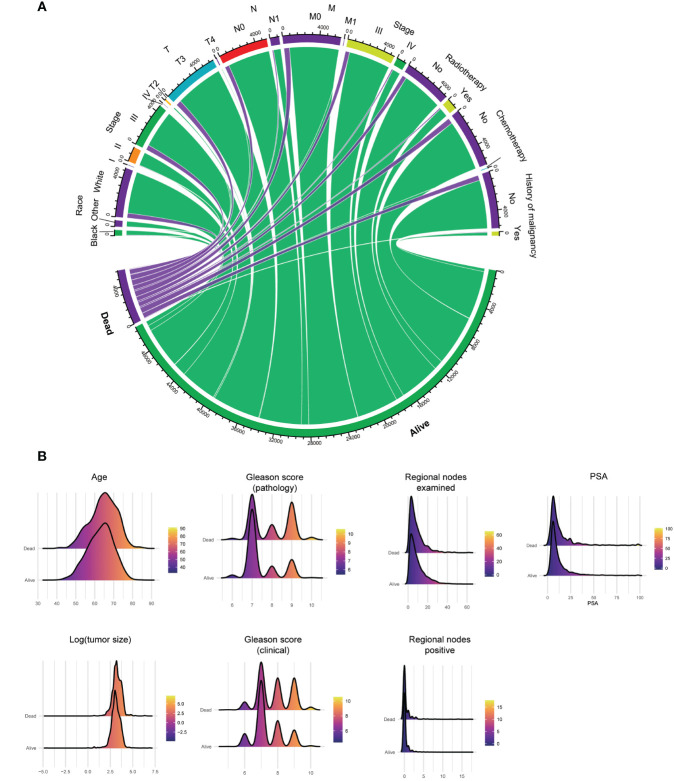
Clinical data’s visualization of postoperative survival in patients with advanced prostate adenocarcinoma (8140/3) and receiving surgery, including categorical **(A)** and numerical **(B)** clinical features. PSA, prostate-specific antigen.

**Figure 4:**
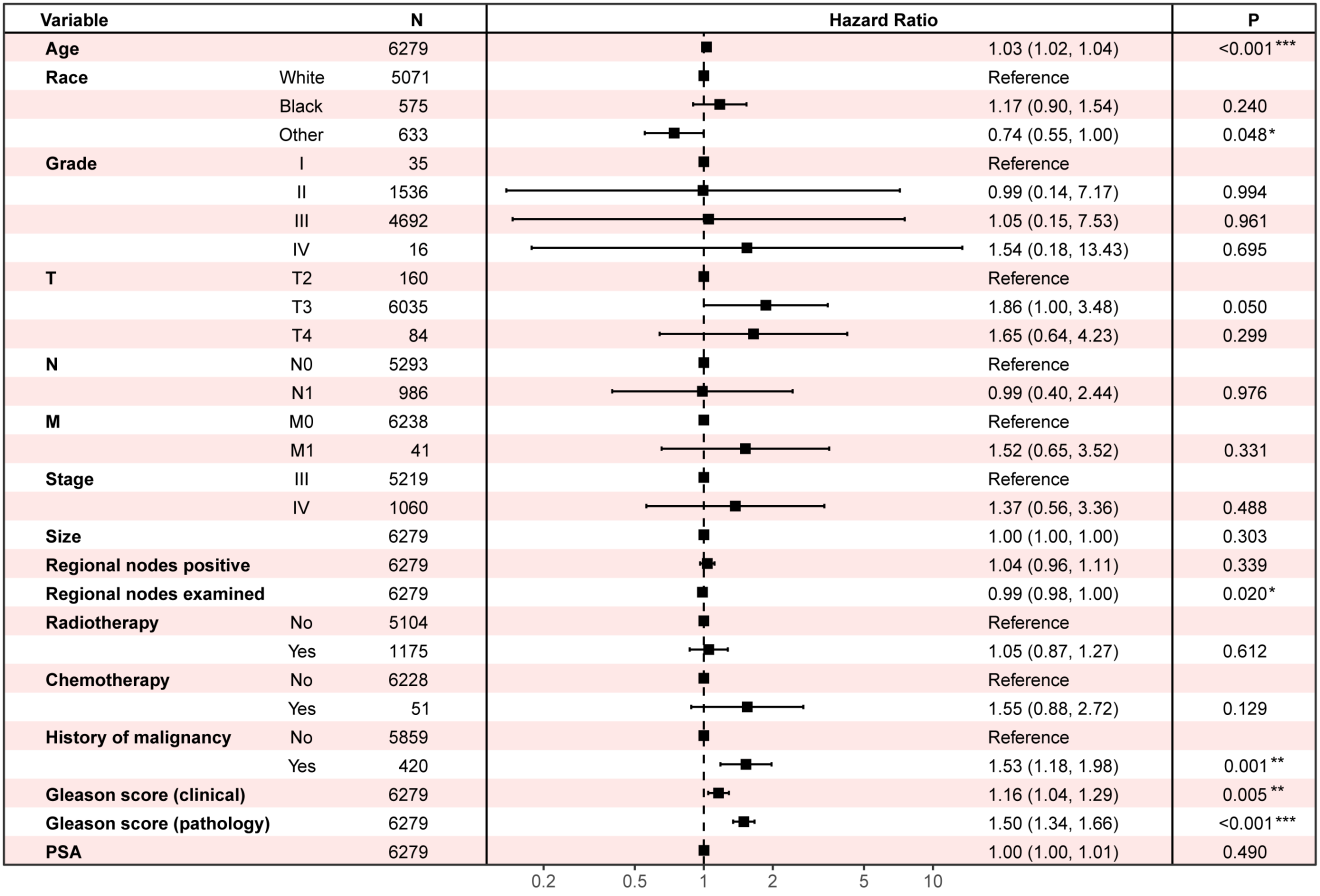
Multivariate Cox regression to find the influential factors of advanced prostate adenocarcinoma (8140/3) patients’ overall survival after surgery. PSA, prostate-specific antigen.

Two cohorts are carried in Cox regression to identify the protective and dangerous factors of advanced prostate adenocarcinoma (8140/3) after surgery. The visualization of patients’ clinical data is shown in **Figure 3**. After analysis, age (HR 1.03, 95% CIs 1.02 - 1.04, P < 0.001), history of malignancy (HR 1.53, 95% CIs: 1.18 - 1.98, P = 0.001), clinical Gleason score (HR 1.16, 95% CIs: 1.04 - 1.29, P = 0.005) and pathological Gleason score (HR 1.50, 95% CIs: 1.34 - 1.66, P < 0.001) tend to dangerous factors. And other race (HR 0.74, 95% CIs: 0.55 - 1.00, P = 0.048), regional nodes examined (HR 0.99, 95% CIs: 0.98 - 1.00, P = 0.020) tend to be protective factors (**Figure 4**).

## Discussion

PC is the most common male malignant tumor and the second most fatal tumor, with 20% progressing to potentially lethal illness (12). PC patients with low malignant potential or indolent disease typically receive active surveillance regimens, patients with localized disease tend to get radiotherapy and RP surgery, and patients with aggressive or metastatic PC usually undergo a combination of several ADT-based therapies such as hormonal therapy, radiotherapy, chemotherapy, and immunotherapy (12).

Trauma, bleeding, and survival benefit or not, are the main reasons that there has been controversy over whether advanced PC should undergo surgical treatment in the past. However, with the boom of treatment like minimally invasive surgical therapy, individuals with advanced PC are no longer confined to ADT (13). Surgery, and radiation, with or without ADT, are increasingly being used to treat advanced PC patients, particularly locally advanced PC patients (10, 14). John F. Ward et al. found the respective cancer-specific survival (CSS) rates of 5652 T3 advanced PC patients after RP were 95%, 90% and 79%, and the complications and incontinence rate was similar to T2 PC patients (15). Chao-Yu Hsu et al. reported that in 235 T3a PC patients after RP, the OS of 5 and 10 years reached 95.9% and 77.0%, and CSS was 98.7% and 91.6% respectively. They also observed 23.5% cT3a PC patients were clinically over-staged (pT2), which might cause them lose the surgery chance as a result (16). After analyzing 1093 cT4 PC patients, Peter A. S. Johnstone et al. noticed that T4 PC patients who received RP treatment had the highest 5-year OS and relative survival rate when compared to those who got therapy ADT, radiotherapy or ADT and radiotherapy combination treatment (17). Ryan K Berglund et al. also believed neoadjuvant goserelin acetate and flutamide therapy followed by RP was feasible and might be an alternative to a strategy of combined radiation and ADT (18). RP has also been shown to improve survival in PC individuals with lymph nodes and distant metastases. Thomas Steuber et al. noted that among 158 localized PC patients with lymph node metastasis, patients after RP had longer PFS compared with unoperated patients (P = 0.005) (19). Jutta Engel et al. also held the view that lymph node positive patients with full RP had better survival than patients with abandoned RP, and that RP was a significant independent predictor of survival (P < 0.0001) (20). After identifying 8185 patients, Stephen H. Culp et al. thought metastatic PC patients having RP (67.4% in OS and 75.8% in disease-specific survival, DSS) or brachytherapy (52.6 in OS and 61.3% in DSS) had substantially higher than no surgery or radiation therapy patients (22.5% in OS and 48.7% in DSS, respectively) (P < 0.001) (21). Axel Heidenreich also observed in adequately-chosen males with metastatic PC who react well to neoadjuvant ADT, CP or RP is a viable option (9).

The present quandary is determining which advanced PC patients may benefit from surgery and what their unique prognosis is (10). Accurately estimating an advanced PC patient’s prognosis is not only a worry for patient and his families, but it is also a potential reference for clinical decision-making. For example, in the clinical scenario that we imagine, doctors can utilize DeepPC to estimate the difference or benefit in OS probability between performing surgery and not doing, when talking about an advanced prostate cancer patient. Besides, some concerns are heightened by the fact that the existing evidence of advanced PC patients’ surgical benefit is still retrospective, with no prospective randomized controlled clinical studies. In light of this condition, we attempt to develop a model to forecast advanced PC patients’ survival.

At present, the most often used technique for constructing prediction models is based on CPH, which investigates the relationship between variables and survival time and provides recommendations on their HR based on a linear hypothesis. As a semi-parametric and linear model, it may not be suitable to predict survival for limited precision. Therefore, the DeepSurv algorithm, developed by Eu-Tteum Baek and colleagues, has been taken a good use of completing this study (22, 23). DeepSurv converges deep neural network and CPH regression, and it can find out about the complex and nonlinear relationships between prognostic clinical variables and an individual’s probability of mortality in true world, which has shown huge potential on medical field (24–26). Our previous studies have also demonstrated DeepSurv may outperform CPH in predicting tumor patients’ survival (27, 28). Therefore, we construct survival models using both CPH and DeepSurv algorithm this time, using all variables collected or LASSO to filter potential predictive clinical features, and chose the better one to serve as the final model.

In this study, we include 6380 diagnosed with advanced (stage III-IV) PC patients who got surgery from SEER database. After random sampling, 4466 samples (70% of total) in train cohort are used to construct prediction model to forecast their prognosis, and 1914 samples (30% of total) in test cohort are utilized to validate this model further. The model using all collected clinical features as predictors and based on neural network algorithm performs best, which scores 0.7058 AUC (95% CIs, 0.7021-0.7068) in train cohort and 0.6925 AUC (95% CIs, 0.6906-0.6956) in test cohort. We then package it into a Windows 64-bit software.

There is currently no predictive model for postoperative survival in patients with advanced PC. We reviewed other postoperative prostate cancer prediction models designed for non-advanced PC. Enchong Zhang et al. developed a PC prognostic model based on six DNA methylation sites, which scored 0.823-0.891 AUC. But their model lacks validation on independent datasets (29). Linda G W Kerkmeijer et al. analyzed 3383 localized PC patients, and built a model to predict their DSS before treatment. The C-statistic of their model was 0.78 (95% CIs: 0.74 - 0.82) (30). Zezhen Liu et al. constructed an immune-related biomarker-based risk model to predict PC prognosis, which got 0.749-0.804 AUC (31). (**Supplementary Table 4**) These findings suggest that the use of biomarkers such as gene expression may improve the accuracy of PC prognostic prediction.

The AUC of DeepPC is about 0.7, showing predictive value but moderate. On the one hand, it could be due to the large difference in prognosis for advanced prostate cancer, while on the other, it could be due to diverse surgical procedures. Because the SEER database lacks extensive descriptions of surgical procedures and biomarker information, the impacts discussed above are not included in this analysis, which limit model’s performance. Besides, models in this study are established only based on SEER data, and more prospective and multi-center data may better train and validate DeepPC. We and other researchers can investigate upgrading these metrics in future study in order to increase DeepPC performance.

## Conclusion

Patients with advanced prostate cancer may benefit from surgery. In order to forecast their overall survival, we first build a clinical features-based prognostic model. This model is accuracy and may offer some reference on clinical decision making.

## Data availability statement

The datasets used and analyzed during the current study are available from the corresponding authors on reasonable request.

## Ethics statement

Ethical approval was not required for the study involving humans in accordance with the local legislation and institutional requirements. Written informed consent to participate in this study was not required from the participants or the participants’ legal guardians/next of kin in accordance with the national legislation and the institutional requirements.

## Author contributions

SL: Conceptualization, Data curation, Formal Analysis, Methodology, Writing – original draft, Writing – review & editing. SC: Data curation, Formal Analysis, Methodology, Writing – original draft. JH: Data curation, Methodology, Writing – original draft. ZL: Data curation, Formal Analysis, Methodology, Writing – original draft. ZS: Data curation, Formal Analysis, Methodology, Writing – original draft. KZ: Formal Analysis, Methodology, Writing – original draft. JJ: Conceptualization, Investigation, Writing – original draft, Writing – review & editing. WL: Conceptualization, Data curation, Formal Analysis, Investigation, Methodology, Software, Writing – original draft, Writing – review & editing. YP: Conceptualization, Methodology, Project administration, Supervision, Writing – original draft, Writing – review & editing.

## Supplementary material

Supplementary Table 1 Coefficients of clinical features in least absolute shrinkage and selection operator (LASSO) regression.

Supplementary Table 2 The value of the mean and standard deviation of the variable.

Supplementary Table 3 Numerical encodings of categorical variables.

Supplementary Table 4 Model performance in predicting prognosis of patients with non-advanced prostate cancer in part previous literature.

Supplementary Figure 1
**(A)** The change of coefficients in least absolute shrinkage and selection operator (LASSO) Cox regression when used to filter clinical features. **(B)** the training curves of neural networks when using variables filtered by LASSO. **(C)** the training curves of neural networks when using all collected clinical features.

Supplementary Figure 2 The parameters of survival predictive tool for advanced prostate cancer patients after surgery (DeepPC).

Supplementary Figure 3 Python version of survival predictive tool for advanced prostate cancer patients after surgery (DeepPC), which is suitable for batch calculations of large-scale population.

Supplementary File 1 Python codes used to train predictive tool for advanced prostate cancer patients after surgery (DeepPC).

Supplementary File 2 The predictive tool for advanced prostate cancer patients after surgery (DeepPC) (windows version). Linkage: https://drive.google.com/file/d/1VXp4JKPxniSWe-F0k9tmGW6D8Oe1Agkb/view?usp=sharing
