# Supplementary material for: Develop prediction model to help forecast advanced prostate cancer patients’ prognosis after surgery using neural network
Source: Front Endocrinol (Lausanne). 2024 Mar 21;15:1293953. doi: 10.3389/fendo.2024.1293953 (PMC10991752; doi:10.3389/fendo.2024.1293953)
Supplement: Supplementary Table 1 — Coefficients of clinical features in least absolute shrinkage and selection operator (LASSO) regression. [file Table_1.docx]

| Supplement Table 1. Coefficients of clinical features in least absolute shrinkage and selection operator (LASSO) regression. | |
| --- | --- |
| Clinical features | Coefficient |
| Age | 0.02 |
| Race | 0.00 |
| Grade | 0.00 |
| Pathology | 0.00 |
| T | 0.00 |
| N | 0.00 |
| M | 0.04 |
| Stage | 0.12 |
| Size | 0.00 |
| Regional nodes positive | 0.00 |
| Regional nodes examined | 0.00 |
| Radiotherapy | 0.00 |
| Chemotherapy | 0.08 |
| History of malignancy | 0.20 |
| Gleason score (clinical) | 0.16 |
| Gleason score (pathology) | 0.37 |
| PSA | 0.00 |
| Gleason score (clinical), composed of needle core biopsy or transurethral resection of the prostate specimens. Gleason score (pathology), composed of prostatectomy specimens. PSA, prostate-specific antigen. | |
