# Supplementary material for: Develop prediction model to help forecast advanced prostate cancer patients’ prognosis after surgery using neural network
Source: Front Endocrinol (Lausanne). 2024 Mar 21;15:1293953. doi: 10.3389/fendo.2024.1293953 (PMC10991752; doi:10.3389/fendo.2024.1293953)
Supplement: Supplementary Table 2 — The value of the mean and standard deviation of the variable. [file Table_2.docx]

| Supplement Table 2. The value of the mean and standard deviation of the variable | | |
| --- | --- | --- |
|  | Mean | Standard deviation |
| Age | 63.28 | 6.84 |
| Size | 26.96 | 31.33 |
| Regional nodes positive | 0.32 | 1.05 |
| Regional nodes examined | 8.96 | 7.60 |
| PSA | 12.23 | 12.78 |
| Gleason score (clinical) | 7.44 | 0.89 |
| Gleason score (pathology) | 7.51 | 0.86 |
| PSA, prostate-specific antigen. Gleason score (clinical), composed of needle core biopsy or transurethral resection of the prostate specimens. Gleason score (pathology), composed of prostatectomy specimens. | | |
