# Supplementary material for: Develop prediction model to help forecast advanced prostate cancer patients’ prognosis after surgery using neural network
Source: Front Endocrinol (Lausanne). 2024 Mar 21;15:1293953. doi: 10.3389/fendo.2024.1293953 (PMC10991752; doi:10.3389/fendo.2024.1293953)
Supplement: Supplementary Table 3 — Numerical encodings of categorical variables. [file Table_3.docx]

| Supplement Table 3. Numerical encodings of categorical variables. | |
| --- | --- |
|  | Encoding |
| Race |  |
| White | 0 |
| Black | 1 |
| Other | 2 |
| Grade |  |
| I | 0 |
| II | 1 |
| III | 2 |
| IV | 3 |
| Pathology | |
| 8140/3: Adenocarcinoma, NOS | 0 |
| 8201/3: Cribriform carcinoma, NOS | 1 |
| 8246/3: Neuroendocrine carcinoma, NOS | 2 |
| 8255/3: Adenocarcinoma with mixed subtypes | 3 |
| 8480/3: Mucinous adenocarcinoma | 4 |
| 8481/3: Mucin-producing adenocarcinoma | 5 |
| 8490/3: Signet ring cell carcinoma | 6 |
| 8500/3: Infiltrating duct carcinoma, NOS | 7 |
| 8550/3: Acinar cell carcinoma | 8 |
| 8574/3: Adenocarcinoma with neuroendocrine differentiation | 9 |
| T |  |
| T1 | 0 |
| T2 | 1 |
| T3 | 2 |
| T4 | 3 |
| N |  |
| N0 | 0 |
| N1 | 1 |
| M |  |
| M0 | 0 |
| M1a | 1 |
| M1b | 2 |
| M1c | 3 |
| Stage |  |
| III | 0 |
| IV | 1 |
| Radiotherapy | |
| No | 0 |
| Yes | 1 |
| Chemotherapy | |
| No | 0 |
| Yes | 1 |
| History of malignancy | |
| No | 0 |
| Yes | 1 |
| Grade I, well differentiated; Grade II, moderately differentiated; Grade III, poorly differentiated; Grade IV, undifferentiated, anaplastic. NOS, not otherwise specified. | |
