# Supplementary material for: Develop prediction model to help forecast advanced prostate cancer patients’ prognosis after surgery using neural network
Source: Front Endocrinol (Lausanne). 2024 Mar 21;15:1293953. doi: 10.3389/fendo.2024.1293953 (PMC10991752; doi:10.3389/fendo.2024.1293953)
Supplement: Supplementary Table 4 — Model performance in predicting prognosis of patients with non-advanced prostate cancer in part previous literature. [file Table_4.docx]

| Supplement Table 4. Model performance in predicting prognosis of patients with non-advanced prostate cancer in part previous literature | | |
| --- | --- | --- |
|  | Predictive variables | Performance |
| Enchong Zhang et al | Six DNA methylation sites | 0.823-0.891 AUC |
| Linda G W Kerkmeijer et al | Clinical features | 0.78 C-statistic |
| Liu et al | Immune-related biomarkers | 0.749-0.804 AUC |
| AUC, area under receiver operating characteristic curve. | | |
