# Supplementary figures and images for: Develop prediction model to help forecast advanced prostate cancer patients’ prognosis after surgery using neural network
Source: Front Endocrinol (Lausanne). 2024 Mar 21;15:1293953. doi: 10.3389/fendo.2024.1293953 (PMC10991752; doi:10.3389/fendo.2024.1293953)

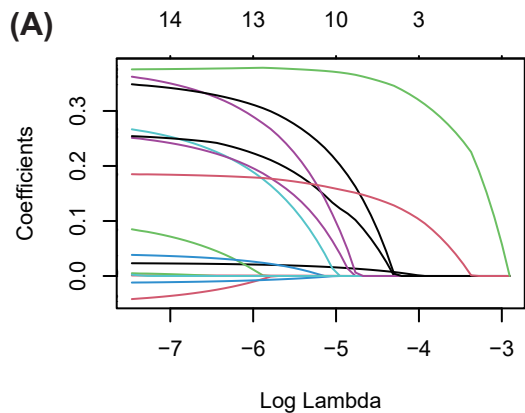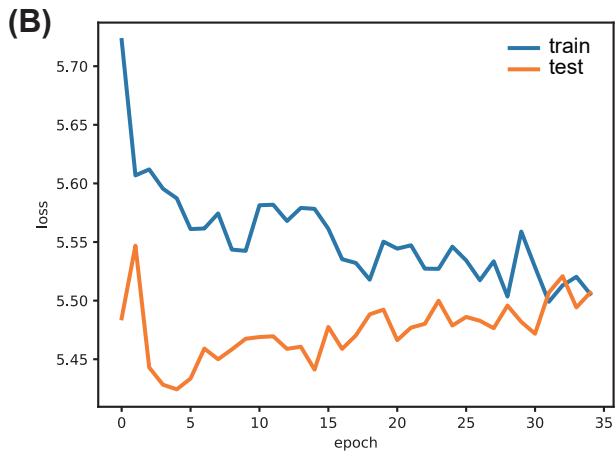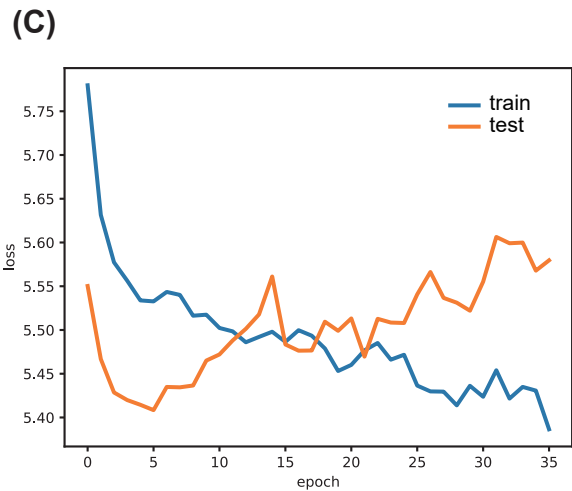

Supplement: Supplementary Figure 1 — (A) The change of coefficients in least absolute shrinkage and selection operator (LASSO) Cox regression when used to filter clinical features. (B) the training curves of neural networks when using variables filtered by LASSO. (C) the training curves of neural networks when using all collected clinical features. [file Image_1.pdf]
