# Supplementary material for: Develop prediction model to help forecast advanced prostate cancer patients’ prognosis after surgery using neural network
Source: Front Endocrinol (Lausanne). 2024 Mar 21;15:1293953. doi: 10.3389/fendo.2024.1293953 (PMC10991752; doi:10.3389/fendo.2024.1293953)
Supplement: Supplementary Figure 2 — The parameters of survival predictive tool for advanced prostate cancer patients after surgery (DeepPC). [file Image_2.pdf]

```
OrderedDict([('net.0.linear.weight', tensor([[-0.7447, -0.4986,  0.2519, -0.1189, -0.2790, -0.6699, -
0.1789, -0.8765,
           0.0215, -0.8449,  0.1893, -1.1500,  0.1007, -1.0178, -0.9523,  0.1394,
          -0.3600],
         [-0.6126,  0.0858, -0.5234,  0.4545, -0.0173, -0.4664, -0.1712, -1.0568,
           0.5795, -0.2035, -0.5308, -0.5393, -0.2722,  0.0563, -0.5082, -0.0760,
           0.0506],
         [-0.8109,  0.8371, -0.5144,  0.3084, -0.3429, -0.3054,  0.0030,  0.6991,
           0.5770, -1.0432, -0.1211, -0.5506, -0.2731,  0.4715, -0.5812, -0.6158,
           0.1854],
         [ 0.1983,  0.0577,  0.5608,  0.5755,  0.2751,  0.8021, -0.4880,  0.3826,
           0.2782,  0.1395,  0.7708,  0.3570,  0.3937,  0.5217, -0.0648, -0.7490,
           0.0465],
         [-0.4330,  0.2381,  0.6250, -0.2272,  0.5158,  0.5908, -0.3814,  0.7194,
           0.3988,  0.4550, -0.1366,  0.2341,  0.1600, -0.5447, -0.3550,  0.6846,
          -0.8815],
         [-0.5834,  0.3688,  1.2763, -0.2926,  0.5820, -0.0683, -0.6973, -0.7629,
          -0.2061, -0.3386,  0.3747,  0.4700, -0.3492, -0.7644, -0.3953, -0.4496,
           0.1247],
         [ 0.3295,  0.8042,  0.4391,  0.1758,  0.1368, -0.2902,  0.0537, -0.8408,
          -0.5155,  0.3590, -0.1579,  0.5241,  0.3509,  0.1298,  0.0914,  0.3039,
           0.8331],
         [ 0.8729, -0.4908,  0.9545,  0.1751,  0.1079,  0.6962,  0.2608, -0.4530,
          -0.0890,  0.1101,  0.4531, -0.5583, -0.1452, -0.1066, -0.0069, -0.0634,
           0.3870],
         [ 0.2063, -0.4092,  0.4970, -0.1606,  0.1833, -0.1478,  0.4519,  0.6599,
           0.5174, -0.1042, -0.0580, -0.1198,  0.2287,  1.0959, -0.1207,  0.9143,
           0.5185],
         [-0.4938,  0.5436, -0.1824,  0.6972,  0.2562,  0.6049, -0.6583,  0.3679,
           0.7036, -0.2788,  0.5675, -0.3669,  0.5881,  0.5832, -0.5180, -0.5549,
```

-0.0261],
[0.9403, -0.0702, 0.5434, -0.4098, 0.3160, -0.7794, -0.2406, -0.4451,
0.1281, 0.1287, 0.6899, 0.3127, -0.7614, -0.1594, 0.1077, -0.7003,
-0.4116],
[0.1994, -0.0999, -0.4301, -0.1474, -0.5100, 0.7444, 0.7048, 0.1041,
-0.0405, 0.2984, 0.0896, -0.2999, 0.7122, 0.3484, -0.3487, -0.7819,
-0.9828],
[-0.1200, 0.2544, 0.5726, 0.2675, 2.0987, 0.2671, 0.3645, 0.6070,
0.5796, -0.1594, 0.0906, 0.1802, 0.8669, 0.2529, 0.7192, 0.5805,
0.2311],
[0.3496, 0.0637, 0.4335, 0.6486, -0.2527, -1.5039, -0.3171, -0.8758,
0.2624, 0.3756, -0.4803, 0.7229, 0.1089, 0.0980, 0.4198, 0.7495,
-0.9591],
[-0.1218, 0.0558, 0.3614, -0.1627, 0.3600, 0.6226, 0.7070, -0.4497,
1.6467, 0.4030, -0.9060, 0.0590, 0.7390, 0.4431, -0.0410, -0.0324,
0.1656],
[-0.1220, 0.3413, -0.1856, 0.3583, 0.3199, -0.6510, -0.1144, -0.5269,
-0.7408, 0.1345, -0.5436, 1.0930, 0.1504, -0.9884, -0.2871, -0.5943,
0.0805]])), ('net.0.linear.bias', tensor([0.1851, -0.3764, -0.0653, 0.4944, 0.3187, 0.4780, -0.0147, 0.0067,
0.4276, -0.0151, 0.1605, 0.0015, 0.2046, -0.1493, 0.3108, -0.0857])), ('net.0.batch_norm.weight', tensor([0.7600, 1.2422, 1.3443, 0.8009, 0.8132, 1.0471, 0.8968, 0.7552, 1.2046,
1.4159, 0.7645, 1.1695, 1.0937, 1.1414, 1.1660, 0.9408])), ('net.0.batch_norm.bias', tensor([0.4256, -0.3075, -0.3042, 0.3330, -0.0265, 0.4335, 0.2966, -0.2278,
-0.2216, -0.5715, 0.2388, 0.0198, -0.4362, -0.6413, -0.0453, -0.1977])), ('net.0.batch_norm.running_mean', tensor([0.5511, 0.0564, 0.1748, 2.3226, 2.6704, 3.6779, 1.2730, 1.6167, 1.6304,
0.8481, 1.6459, 0.0606, 5.4227, 0.6994, 1.7210, 0.7565])), ('net.0.batch_norm.running_var', tensor([0.6292, 0.1470, 0.3215, 1.7519, 1.8558, 1.5909, 1.4901, 1.1787, 1.8285,

1.2622, 1.3915, 0.0730, 2.8019, 1.3549, 2.8107, 0.6353])), ('net.0.batch_norm.num_batches_tracked', tensor(54)), ('net.1.linear.weight', tensor([[2.3075e-01, -4.3206e-01, -6.3933e-01, -3.3191e-01, -2.6892e-01,
1.4950e-01, 2.5402e-01, 3.6943e-01, 1.9554e-01, -9.4378e-01,
-5.1483e-02, -5.6582e-01, 1.1876e+00, 3.9786e-01, -1.8916e-01,
-2.6807e-01],
[7.1674e-01, 2.7836e-01, -2.1687e-01, -1.9965e-01, 6.0149e-01,
-9.9053e-03, -1.3186e+00, 8.0772e-02, -5.4364e-01, -5.7174e-01,
-6.2096e-01, -1.2024e-01, -1.3415e-01, -4.1915e-01, -9.6912e-01,
2.8451e-01],
[1.3149e+00, 7.7082e-01, -5.8785e-01, -3.6791e-01, -1.3363e-01,
8.6214e-01, -6.0864e-02, 9.1369e-01, 6.7461e-01, -1.0332e-01,
-1.2633e-01, -6.5262e-03, 1.0310e-01, -6.3986e-01, 2.8552e-02,
-1.4449e-01],
[4.4549e-01, -4.2723e-01, 3.8569e-01, -2.8025e-01, 1.6347e-01,
4.8630e-01, -6.8283e-02, -4.7527e-01, 1.3294e-01, -6.9691e-01,
-5.2269e-01, -2.3432e-02, -2.5358e-01, -7.5882e-01, -7.8222e-01,
-9.0246e-02],
[1.2446e-01, 1.6352e-01, 3.0813e-02, -1.6866e-01, 1.2229e-01,
3.3289e-01, -1.5604e-01, -1.5059e-02, -7.9836e-01, 3.1270e-02,
-4.3058e-01, 8.7848e-01, 3.4387e-02, 1.1736e-01, -3.8445e-01,
5.9298e-01],
[5.2198e-01, -2.4546e-01, -6.7202e-01, -1.7125e-01, 1.1162e-02,
2.0449e-01, -3.6864e-01, -1.6476e-01, -8.0097e-01, -1.2452e-02,
-1.3868e-01, -2.0281e-01, -6.5014e-01, -1.0185e+00, 4.7861e-01,
-2.5765e-01],
[-6.5098e-04, -4.1566e-01, -7.4157e-01, 2.9505e-02, 5.3125e-01,
2.3788e-01, 2.9679e-01, -2.5687e-01, -3.2716e-01, -8.1928e-01,
2.2726e-01, 7.4688e-01, -6.8612e-01, -6.3620e-01, -5.2363e-01,
1.9849e-01],

```
[ 3.0612e-01,  3.6955e-02, -6.1538e-01, -8.5519e-01,  2.1434e-01,
 -9.9344e-01,  2.7658e-01,  4.8744e-01,  6.6413e-01, -6.0532e-02,
 -9.3591e-01, -8.2956e-02,  3.8686e-01, -6.1918e-02,  4.0006e-01,
  2.8478e-01],
[-1.0256e-01, -6.2660e-01, -8.3878e-01, -2.3352e-01,  1.3045e-01,
 -9.8282e-01, -4.5471e-01,  4.3850e-01,  6.9187e-01,  4.4460e-03,
  2.3141e-01, -2.8014e-01, -2.1861e-02,  7.1484e-02, -9.2901e-02,
 -7.0013e-01],
[ 8.3440e-01,  8.7904e-01,  6.8369e-01, -5.7788e-01,  1.3769e-01,
  9.2802e-01, -6.5653e-01, -1.7229e-01,  4.2836e-02,  1.2171e+00,
  5.1965e-01,  7.3714e-02,  1.1455e-01,  6.7472e-02, -1.7403e-01,
 -2.3207e-01],
[-2.4362e-01, -7.4902e-01, -4.1501e-01, -2.4607e-01, -5.7661e-01,
 -3.7872e-01,  1.0276e-01, -6.0083e-01, -6.2802e-01,  5.8679e-02,
  1.2179e-01,  1.0061e+00, -5.6822e-01,  4.3984e-01, -3.5531e-02,
 -7.8304e-01],
[-3.5465e-03,  8.7900e-01,  6.9158e-01,  4.8707e-01,  8.7755e-01,
  3.7131e-01, -3.9168e-01, -5.7828e-02,  7.5023e-02,  2.3517e-02,
 -7.6617e-01, -2.4848e-01, -2.5785e-01,  6.6064e-02, -2.6731e-01,
  2.8054e-01],
[-1.4128e-02,  3.2507e-01,  4.3041e-01,  1.1545e+00,  4.2605e-01,
  2.9350e-01,  5.0027e-01,  4.6643e-01,  1.2620e-01,  8.7628e-01,
 -1.0875e+00, -1.9032e-01, -1.4980e-01,  1.9139e-01, -4.0289e-01,
  1.4654e-01],
[-2.3870e-01,  4.6990e-01,  1.3575e+00,  3.4782e-01,  5.5512e-01,
  7.3737e-01, -1.5637e-01,  8.1022e-02,  7.5668e-01,  5.0689e-01,
 -2.3708e-01,  8.2265e-01,  2.4206e-01, -4.7272e-01, -1.0331e-01,
 -1.0619e-01],
[-2.0192e-01,  3.3303e-01,  3.6479e-01, -2.3320e-01, -2.3521e-01,
 -1.1820e-01,  2.9180e-01, -6.2794e-01, -1.6427e-01,  5.2033e-01,
```

-6.5988e-01, -3.2094e-01, 7.6659e-01, 7.8055e-01, -5.5518e-01,
-3.8499e-02],
[-1.4771e-01, -2.9396e-01, -1.3233e-01, 1.2595e-01, 6.8095e-01,
-3.0167e-01, -6.9172e-01, -5.4369e-01, 4.4820e-01, 4.0899e-01,
-8.0927e-01, 6.7270e-02, 1.7134e-01, 5.3818e-01, 8.3250e-01,
-6.3475e-01]])), ('net.1.linear.bias', tensor([0.3430, 0.6069, 0.3765, 1.2221, -0.6915, 0.5283, 0.3785, 0.0546,
0.5508, -0.9211, 0.1475, 0.2199, -0.0668, -0.7984, 0.3575, 0.2832])), ('net.1.batch_norm.weight', tensor([0.4112, 0.8352, 0.6664, 0.5464, 0.6683, 0.1455, 0.6447, 0.6225,
0.7801, 1.0242, 0.6286, -0.0707, 0.3464, 0.7634, 0.4303, 0.9505])), ('net.1.batch_norm.bias', tensor([0.1294, 0.2502, 0.3621, -0.3235, 0.3458, -0.2002, 0.2276, 0.6174,
-0.6606, -0.0313, 0.6180, -0.0117, 0.4633, -0.1625, -0.3369, 0.0345])), ('net.1.batch_norm.running_mean', tensor([1.5381, 1.6109, 1.3405, 2.6556, 0.4382, 2.5199, 2.6188, 0.6643, 1.2481,
0.7977, 1.1442, 0.6207, 0.6377, 0.6614, 0.4565, 0.8753])), ('net.1.batch_norm.running_var', tensor([3.9098, 2.3099, 2.0196, 2.1564, 1.2625, 3.4703, 3.3666, 1.7306, 2.4739,
6.5873, 2.3690, 3.8553, 3.4959, 6.1394, 2.0500, 3.2574])), ('net.1.batch_norm.num_batches_tracked', tensor(54)), ('net.2.weight', tensor([[-1.5484e-02, -2.4352e-01, -8.5190e-02, -8.8558e-02, -7.9590e-02,
2.3533e-04, -9.0709e-02, 1.1409e-01, 1.5614e-01, -2.3052e-01,
-1.0381e-01, 4.2805e-02, 2.2121e-02, -7.8982e-02, 1.3303e-02,
2.4109e-01]]))])
