# Supplementary material for: Develop prediction model to help forecast advanced prostate cancer patients’ prognosis after surgery using neural network
Source: Front Endocrinol (Lausanne). 2024 Mar 21;15:1293953. doi: 10.3389/fendo.2024.1293953 (PMC10991752; doi:10.3389/fendo.2024.1293953)
Supplement: Supplementary Figure 3 — Python version of survival predictive tool for advanced prostate cancer patients after surgery (DeepPC), which is suitable for batch calculations of large-scale population. [file Image_3.pdf]

jupyter DeepProstate (New)

Logout

File Edit View Insert Cell Kernel Widgets Help 可信 Python 3 (ipykernel)

运行 代码

Prediction

In [58]:

```
time_test0, status_test0 = y_train[0], y_train[1]
surv_cox0 = model.predict_surv_df(x_train)
surv_cox0
```

Out[58]:

	0	1	2	3	4	5	6	7	8	9	...	12249	12250	12251	12252	122
**duration**																
**1**	0.999574	0.999590	0.999644	0.999845	0.999441	0.999507	0.999850	0.999864	0.999744	0.999132	...	0.999637	0.999877	0.999577	0.999817	0.9997
**2**	0.999232	0.999262	0.999360	0.999722	0.998993	0.999113	0.999731	0.999755	0.999540	0.998438	...	0.999347	0.999779	0.999239	0.999671	0.9995
**3**	0.998805	0.998852	0.999004	0.999567	0.998434	0.998619	0.999581	0.999619	0.999284	0.997569	...	0.998984	0.999656	0.998815	0.999487	0.9992
**4**	0.998292	0.998358	0.998576	0.999381	0.997761	0.998026	0.999400	0.999455	0.998976	0.996526	...	0.998548	0.999508	0.998306	0.999267	0.9988
**5**	0.997779	0.997865	0.998148	0.999194	0.997088	0.997433	0.999220	0.999291	0.998668	0.995483	...	0.998111	0.999360	0.997797	0.999046	0.9985
**...**	...	...	...	...	...	...	...	...	...	...	...	...	...	...	...	
**115**	0.870285	0.874986	0.890630	0.950902	0.833456	0.851656	0.952415	0.956685	0.920112	0.753637	...	0.888569	0.960802	0.871277	0.942132	0.9137
**116**	0.870285	0.874986	0.890630	0.950902	0.833456	0.851656	0.952415	0.956685	0.920112	0.753637	...	0.888569	0.960802	0.871277	0.942132	0.9137
**117**	0.870285	0.874986	0.890630	0.950902	0.833456	0.851656	0.952415	0.956685	0.920112	0.753637	...	0.888569	0.960802	0.871277	0.942132	0.9137
**118**	0.866537	0.871364	0.887432	0.949416	0.828753	0.847419	0.950974	0.955370	0.917736	0.747045	...	0.885314	0.959609	0.867556	0.940389	0.9112
**119**	0.866537	0.871364	0.887432	0.949416	0.828753	0.847419	0.950974	0.955370	0.917736	0.747045	...	0.885314	0.959609	0.867556	0.940389	0.9112

119 rows × 12259 columns

In [59]:

```
surv_cox0.to_csv("OS_prediction.csv")
```
