# Supplementary material for: Develop prediction model to help forecast advanced prostate cancer patients’ prognosis after surgery using neural network
Source: Front Endocrinol (Lausanne). 2024 Mar 21;15:1293953. doi: 10.3389/fendo.2024.1293953 (PMC10991752; doi:10.3389/fendo.2024.1293953)
Supplement: Supplementary File 1 — Python codes used to train predictive tool for advanced prostate cancer patients after surgery (DeepPC). [file DataSheet_1.docx]

import torch

import torchtuples as tt

import numpy as np

from sklearn.preprocessing import StandardScaler, LabelEncoder

from sklearn.model_selection import train_test_split

from sklearn.metrics import roc_auc_score

import pandas as pd

from torchtuples.practical import MLPVanilla, accuracy_binary

from pycox.models import CoxPH

from pycox.evaluation import EvalSurv

import matplotlib.pyplot as plt

raw = pd.read_csv("num.csv",na_values="Unknown")

raw.head()

raw.columns

train = raw[raw.Group == "Train"]

train.head()

test = raw[raw.Group == "Test"]

test.head()

train_x = train.loc[:,'Age':'PSA']

test_x = test.loc[:,'Age':'PSA']

num_var = ["Age",'Size','Regional.nodes.positive', 'Regional.nodes.examined','PSA','Gleason.C.', 'Gleason.P.']

train_x[num_var].describe()

x_train = train_x.copy()

x_train["Age"] =( x_train["Age"] - 63.279221 ) / 6.836121

x_train["Size"] =( x_train["Size"] - 26.955889 ) / 31.334743

x_train["Regional.nodes.positive"] =( x_train["Regional.nodes.positive"] - 0.316614 ) / 1.054857

x_train["Regional.nodes.examined"] =( x_train["Regional.nodes.examined"] - 8.960591 ) / 7.596325

x_train["PSA"] =( x_train["PSA"] - 12.231348 ) / 12.782991

x_train["Gleason.C."] =( x_train["Gleason.C."] - 7.436632 ) / 0.889980

x_train["Gleason.P."] =( x_train["Gleason.P."] - 7.505374 ) / 0.856615

x_test = test_x.copy()

x_test["Age"] =( x_test["Age"] - 63.279221 ) / 6.836121

x_test["Size"] =( x_test["Size"] - 26.955889 ) / 31.334743

x_test["Regional.nodes.positive"] =( x_test["Regional.nodes.positive"] - 0.316614 ) / 1.054857

x_test["Regional.nodes.examined"] =( x_test["Regional.nodes.examined"] - 8.960591 ) / 7.596325

x_test["PSA"] =( x_test["PSA"] - 12.231348 ) / 12.782991

x_test["Gleason.C."] =( x_test["Gleason.C."] - 7.436632 ) / 0.889980

x_test["Gleason.P."] =( x_test["Gleason.P."] - 7.505374 ) / 0.856615

x_train = x_train.values.astype(np.float32)

x_test = x_test.values.astype(np.float32)

x_train.shape[1]

Y = lambda df_new: (df_new['duration'].values, df_new['event'].values)

y_train = Y(train)

y_test = Y(test)

in_features = x_train.shape[1]

num_nodes = [16,16]

out_features = 1

batch_norm = True

dropout = 0.1

output_bias = False

epochs = 100

callbacks = [tt.callbacks.EarlyStopping(patience=30)]

#callbacks = None

verbose = True

batch_size = 512

net = tt.practical.MLPVanilla(in_features, num_nodes, out_features, batch_norm,

dropout, output_bias=output_bias)

model_cox = CoxPH(net, tt.optim.Adam(lr=0.05)) #cox model on the neural network with Adam optimizer

log_cox = model_cox.fit(x_train, y_train, batch_size, epochs, callbacks, verbose,

val_data =(x_test,y_test), val_batch_size=batch_size)

#Train

time_test0, status_test0 = y_train[0], y_train[1]

surv_cox0 = model_cox.predict_surv_df(x_train) # survival of the train data

eval_cox0 = EvalSurv(surv_cox0, time_test0, status_test0, censor_surv= 'km')

cox_index0 = eval_cox0.concordance_td()

cox_index0

# test

model_cox.compute_baseline_hazards() #baseline hazard

surv_cox = model_cox.predict_surv_df(x_test) # survival of the test data

# evaluate the c-index of the cox model

eval_cox = EvalSurv(surv_cox, y_test[0], y_test[1], censor_surv= 'km')

cox_index = eval_cox.concordance_td()

cox_index # cox_index at all

%matplotlib inline

%config InlineBackend.figure_format = 'svg'

log_cox.to_pandas()[['train_loss', 'val_loss']].plot()

plt.xlabel('epoch')

plt.ylabel('loss')

plt.savefig('os.pdf', bbox_inches='tight')

plt.show()

model_cox.save_model_weights("os_param.pt")

model_cox.save_net("os_net.pt")

# train

# ATTENTION :x_train, y_train is numpy.ndarray

in_tem = []

model_cox.compute_baseline_hazards()

for i in range(1000):

idx = np.random.choice(len(x_train),size = 500,replace = True)

x_tem = x_train[idx,]

surv_cox_tem = model_cox.predict_surv_df(x_tem) # survival of the test data

eval_cox_tem = EvalSurv(surv_cox_tem, y_train[0][idx], y_train[1][idx], censor_surv= 'km')

c_tem = eval_cox_tem.concordance_td()

in_tem.append(c_tem)

in_mean = np.mean(in_tem)

in_se = np.std(in_tem) / np.power(1000,0.5)

print("Bootstrap Internal validation:")

print("The C-index:{}".format(in_mean))

print("The C-index High 95% CI:{}".format(in_mean + 1.96 * in_se))

print("The C-index Low 95% CI:{}".format(in_mean - 1.96 * in_se))

# Test

ex_tem = []

model_cox.compute_baseline_hazards()

for i in range(1000):

idx = np.random.choice(len(x_test),size = 500,replace = True)

x_tem = x_test[idx,]

surv_cox_tem = model_cox.predict_surv_df(x_tem) # survival of the test data

eval_cox_tem = EvalSurv(surv_cox_tem, y_test[0][idx], y_test[1][idx], censor_surv= 'km')

c_tem = eval_cox_tem.concordance_td()

ex_tem.append(c_tem)

ex_mean = np.mean(ex_tem)

ex_se = np.std(ex_tem) / np.power(1000,0.5)

print("Bootstrap External validation:")

print("The C-index:{}".format(ex_mean))

print("The C-index High 95% CI:{}".format(ex_mean + 1.96 * ex_se))

print("The C-index Low 95% CI:{}".format(ex_mean - 1.96 * ex_se))
